# Integrated Analysis of the Wood Oil from *Xanthocyparis vietnamensis* Farjon & Hiep. by Chromatographic and Spectroscopic Techniques

**DOI:** 10.3390/molecules21070840

**Published:** 2016-06-27

**Authors:** Ophélie Bazzali, Tran Huy Thai, Tran Minh Hoi, Nguyen Sinh Khang, Nguyen Thi Hien, Joseph Casanova, Ange Bighelli, Félix Tomi

**Affiliations:** 1 Université de Corse-CNRS, UMR 6134 SPE, Equipe Chimie et Biomasse, Route des Sanguinaires, 20000 Ajaccio, France; ophelie.bazzali@gmail.com (O.B.); joseph.casanova@wanadoo.fr (J.C.); ange.bighelli@univ-corse.fr (A.B.); 2 Institute of Ecology and Biological Resources, Vietnam Academy of Science and Technology, Cau Giay, Hanoi 10000, Vietnam; thaiiebr@yahoo.com.vn (T.H.T.); tran.minhhoi@yahoo.com (T.M.H.); nskhang@gmail.com (N.S.K.); Nguyenhien333@yahoo.com (N.T.H.)

**Keywords:** *Xanthocyparis vietnamensis*, essential oil composition, nootkatene, 10-*epi*-nor-γ-eudesmen-11-one, 12-hydroxy-isodihydroagarofuran, 11,12,13-tri-nor-eremophil-1(10)-en-7-ol, ^13^C-NMR

## Abstract

In order to get better knowledge about the volatiles produced by *Xanthocyparis vietnamensis,* a species recently discovered in Vietnam, its wood oil has been analyzed by a combination of chromatographic (GC, CC) and spectroscopic (GC-MS, ^13^C-NMR) techniques. Forty components that accounted for 87.9% of the oil composition have been identified. The composition is dominated by nootkatene (20.7%), 11,12,13-tri-nor-eremophil-1(10)-en-7-one (17.2%), γ-eudesmol (5.1%), nootkatone (4.7%), valencene (3.5%) and 13-nor-eremophil-1(10)-en-11-one (2.6%). The structure of two new compounds—10-*epi*-nor-γ-eudesmen-11-one and 12-hydroxy-isodihydroagarofuran—has been elucidated, while 11,12,13-tri-nor-eremophil-1(10)-en-7-ol is reported as a natural product for the first time. The composition of *X. vietnamensis* wood oil varied drastically from those of leaf oils, dominated by hedycaryol (34.4%), phyllocladene (37.8%) or by pimara-6(14)-15-diene (19.4%).

## 1. Introduction

**Figure 1:**
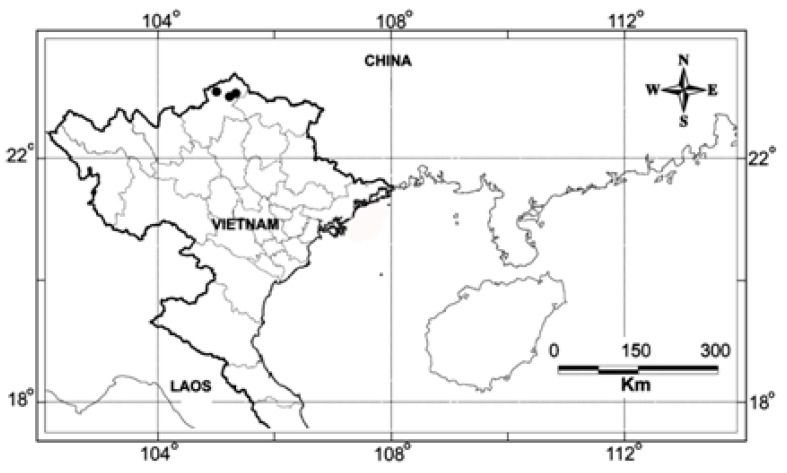
Distribution map of *Xanthocyparis vietnamensis* Farjon & Hiep in Vietnam.

*Xanthocyparis vietnamensis* Farjon & Hiep (Cupressaceae), also known as Vietnamese gold cypress (Vietnamese name: Bách vàng), was discovered in 1999 in the calcareous mountains of Northern Vietnam (Quản Bạ, Hà Giang Province, Figure 1) [1]. Although found since that time in other localities (Hà Giang, Cao Bằng, Tuyên Quang provinces), it grows in a restricted area at an altitude of 1000–1600 m. The estimated number of adult trees is in the range 500–1000 individuals, therefore, *X. vietnamensis* remains among the endangered species [2]. This species has also been added to Group IA of the National List of Rare and Precious Flora and Fauna which prohibits any exploitation. An ex situ conservation program has been initiated and some restoration work undertaken [3]. This species grows associated with conifer species: *Pseudotsuga sinensis*, *Nageia jneiryi*, *Podocarpus pilgeri, Calocedrus macrolepis*, *Taxils chinensis*, *Amentotaxus sp*.

The discovery of this new species led to the proposal of a new genus in 2002, the genus *Xanthocyparis* [4]. From the taxonomic point of view, analysis of 54 characteristics demonstrated the proximity of *Chamaecyparis nootkatensis* (D. Don) Spach with *X. vietnamensis* and therefore the former species has been transferred to the genus *Xanthocyparis* and it is nowadays inventoried as *Xanthocyparis nootkatensis* (D. Don) Farjon & Harder. This denomination was agreed in 2011 by the International Association for Plant Taxonomy [5,6].

*X. vietnamensis* is an evergreen, medium-sized tree, 10–15 m in height, 0.5 m in diameter. Bark is smooth, thin, red or brown-red and fibrous. The originality of this tree is the occurrence, at the adult stage, of two types of leaves (needles and tortoiseshells) [7]. The tree bears both male and female cones, solitary at the top of the branches, coming to maturity in two years [4]. Female cones are nearly globose, 9–11 mm × 10–12 mm, black or dark brown when ripe. Seeds occur in February. Male cones are oval, 2.5–3.5 mm × 2.0–2.5 mm. The wood is odoriferous and of excellent quality and it is used by local craftsmen for house building and house furniture as well as for wood-fired heating [1].

Only two papers have reported on the chemical composition of *X. vietnamensis* essential oil (EO). Three oil samples have been obtained by vapor distillation of fresh leaves collected on trees cultivated in the botanical garden of Edinburgh (Scotland). The essential oil composition was dominated by sesquiterpenes and diterpenes like hedycaryol (1.4%–34.4%), phyllocladene (0.9%–37.8%), germacrene D (3.7%–7.9%) and sandaracopimara-8(14),15-diene (0.7%–8.9%) and it varied substantially from sample to sample. Monoterpenes present at appreciable contents were α-pinene (0.8%–14.9%) and myrcene (0.3%–7.9%) [8]. Another *X. vietnamensis* leaf oil sample isolated from trees growing wild in Vietnam (yield = 0.03% vs. dry material) exhibited a substantially different composition with germacrene D (14.6%), pimara-6(14)-15-diene (19.4%), the unusual cyclohexen-1,5-dienthenyl-3-ene (12.7%) and *epi*-bicyclosesquiphellandrene (6.4%) as main components [9].

The aim of the present work was to get a better knowledge of the volatiles produced by *X. vietnamensis* by analyzing an essential oil sample isolated from the wood of this species. Due to the complexity of this essential oil, analysis was conducted by a combination of chromatographic and spectroscopic techniques.

## 2. Results

Wood from *X. vietnamensis* has been collected in Hà Giang Province (Figure 1) and it produced by water-distillation a pale-yellow essential oil with a yield of 0.20% (v:m).

### 2.1. Analysis of X. vietnamensis Wood Oil by GC(RI), GC-MS and ^13^C-NMR

**Figure 2:**
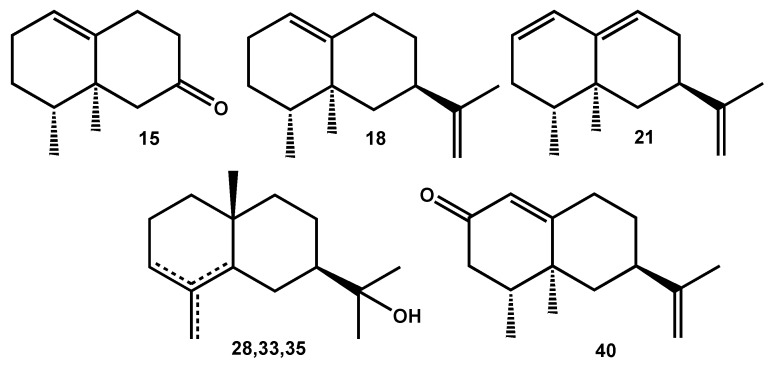
Major components of *X. vietnamensis* wood oil: **15** = 11,12,13-tri-nor-Eremophil-1(10)-en-7-one; **18** = Valencene; **21** = Nootkatene; **28** = γ-Eudesmol; **33** = β-Eudesmol; **35** = α-Eudesmol; **40** = Nootkatone.

The oil sample has been analyzed by GC(RI), GC-MS and ^13^C-NMR without isolation of individual components following a computerized method developed at the University of Corsica [10,11,12]. In total, 28 compounds were identified accounting for 82.8% of the whole composition (Table 1). However, various components present at appreciable contents (up to 2.3%) remained unidentified. In detail and taking in mind that direct identification of a component of an EO by ^13^C-NMR without isolation may be achieved if the compound accounts at least for 0.3%–0.4% of the whole composition [10,11,12], 17 out of the 28 components have been identified by the three techniques, GC(RI), GC-MS and ^13^C-NMR. They belonged to various families of terpenes:

- Monoterpenes bearing the *p*-menthane skeleton: *p*-cymene (**5**), *p*-cymenene (**7**), *p*-cymen-8-ol (**8**), terpinen-4-ol (**9**), α-terpineol (**10**) and carvacrol (**12**);
- Sesquiterpenes bearing the bicyclo[4.4.0]decane skeleton: β-selinene (**17**), valencene (**18**), δ-cadinene (**22**), γ-eudesmol (**28**), τ-muurolol (**30**), β-eudesmol (**33**), α-eudesmol (**35**);
- Sesquiterpenes with substituted six-membered ring: β-elemene (**14**), β-elemol (**23**). It could be pointed out that β-elemene, being identified by NMR at room temperature, is a metabolite of the plant and it is not an artifact resulting from the thermal rearrangement of germacrene A in the GC injector [13];
- Overall, the two major components of the EO—nootkatene (**21**, 20.7%) and 11,12,13-tri-nor-eremophil-1(10)-en-7-one (**15**, 17.2%) bear the eremophilane skeleton (Figure 2).

Seven minor components have been identified by GC(RI) and GC-MS. They are: α-thujene (**1**), sabinene (**2**), myrcene (**3**), 1,4-cineole (**4**), limonene (**6**), carvacryl methyl oxide (**11**) and cadalene (**37**). Finally, and more surprisingly, four compounds, 13-nor-eremophil-1(10)-en-11-one (**25**, 2.6%), τ-cadinol (**29**, 0.7%), α-cadinol (**34**, 1.9%) and nootkatone (**40**, 4.7%) have been identified by GC(RI) and ^13^C-NMR. Matching against commercial and laboratory-made MS libraries did not provide acceptable fit. This probably means that every GC peak does not belong to a unique component. In contrast, the ^13^C-NMR chemical shifts fitted perfectly with those of the reference compounds compiled in our NMR library.

### 2.2. Fractionation of X. vietnamensis Essential Oil

Taking into account that various components remained unidentified and owing to its complexity, the EO was submitted to column chromatography (CC) and 10 fractions were obtained: F1 (hydrocarbon-containing fraction) and F2-F10 (oxygenated compounds-containing fractions). The 10 fractions have been analyzed by GC(RI) and ^13^C-NMR. The occurrence of various terpene hydrocarbons like sabinene (**2**), myrcene (**3**), limonene (**6**) and cadalene (**37**) in fraction F1 was confirmed by NMR. Moreover, two other sesquiterpenes have been identified: α-selinene (**19**) and γ-cadinene (**20**; 0.7% and 1.0% in F1, 0.3% each in EO). Similarly, two compounds were confirmed in fraction F2: carvacryl methyl oxide (**11**; NMR data from our library, 1.0% in fraction F2) and 1,4-cineole (**4**; 6.0% in fraction F2) by comparison of NMR data with those reported by Asakawa et al. [15]. Finally, fraction F2 contained also dehydrojinkoh-eremol (**38**; 2.0% in F2, 0.2% in EO), identified by comparison of NMR data with those reported by Ishihara et al. [16]. The retention index (RI = 1671) is in agreement with that reported by Tajuddin et al. (RI = 1673) [17]. Other components have been identified by GC(RI) and ^13^C-NMR in various fractions of CC: α-terpinyl acetate (**13**), eremophil-9-en-11-ol (**26**), eremoligenol (**27**), δ-cadinol (torreyol) (**31**), valerianol (**32**) and selina-11-en-4α-ol (**36**).

The chromatographic profile as well as the ^13^C-NMR spectrum of fractions F2, F8 and F9 demonstrated that every fraction contained an unidentified major component. Therefore, it was decided to combine column chromatography (CC on silica gel using a gradient of solvents pentane/diethyl ether) and the concept of “extraction” NMR [18,19] to identify the major components of every fraction.

### 2.3. Structure Elucidation of New Natural Compounds

#### 2.3.1. Identification of 11,12,13-Tri-nor-eremophil-1(10)-en-7-ol (**16**)

**Figure 3:**
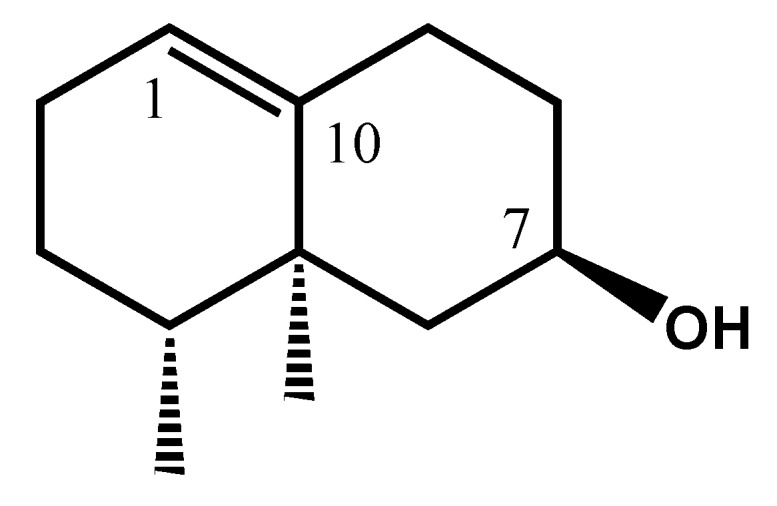
11,12,13-Tri-nor-eremophil-1(10)-en-7-ol (**16**).

Fraction F9 was subjected to CC on silica gel using a gradient of solvents (pentane/Et_2_O). Sub-fraction F9-7 contained a major component **A** (52.5%, 0.4% in EO; RIa/RIp = 1458/2173). The ^13^C-NMR spectrum of this fraction exhibited 12 signals with high intensities. Combination of the information provided by DEPT spectrum (2 C, 3 CH, 5 CH_2_ and 2 CH_3_), and by ^1^H and ^13^C chemical shift values (occurrence of one double bond C=CH and an alcohol function CH-OH), suggested the formula C_12_H_20_O, in agreement with the molecular ion in the MS (M^+^ = 180). Therefore, A is a bicyclic, mono unsaturated tri-nor-sesquiterpene alcohol. Otherwise, 2D NMR experiments, particularly long range proton-carbon connectivities in the HMBC spectrum, suggested the 11,12,13-tri-nor-eremophil-1(10)-en-7-ol structure for **A** (component **16** in Table 1, Figure 3). It could be pointed out that the chemical shifts of seven out of twelve carbons appeared very close to those of 11,12,13-tri-nor-eremophil-1(10)-en-7-one (**15**), one of the major components of the EO. The axial stereochemistry of H7 was ensured by the coupling constant values of its signal (H7, triplet of triplet, *J* = 11.3 and 4.2 Hz). A search in the computerized data library SciFinder revealed that the molecule has been already obtained by Revial et al. [20] in the course of their synthesis of valencenol. It appears that this compound is reported from natural sources for the first time.

#### 2.3.2. Identification of 10-*epi*-Nor-γ-eudesmen-11-one (**24**)

**Figure 4:**
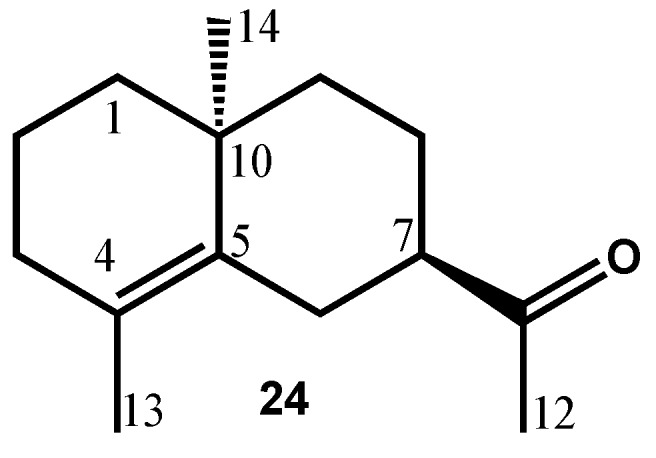
Structure of 10-*epi*-nor-γ-eudesmen-11-one (**24**).

Sub-fraction F2.3, obtained by fractionation of F2 on silica gel, contained a main compound (B, 40.4%, 0.9% in EO; RIa/RIp = 1553/2039). The ^13^C-NMR spectrum of that fraction displayed 14 signals with high intensities that obviously belonged to B (Table 2). DEPT spectra differentiated four quaternary carbons (including a carbonyl carbon and two olefinic carbons), one CH, six CH_2_ and three CH_3_. Therefore, the formula C_14_H_22_O was deduced in agreement with the molecular ion M^+^ = 206 in the MS. Beside the carbonyl function, that belonged to an acetyl group according to chemical shift data, and one double bond, the structure of B contained two rings and therefore it formed a bicyclodecane framework. Two possibilities have been considered: the bicyclo[4.4.0]decane and bicyclo[5.3.0]decane skeletons. Although the ^1^H-NMR spectrum of the fraction was complex, the HSQC spectrum allowed the identification of protons that belonged to B. Then the long range heteronuclear connectivities reported in the Table 2 conducted to the structure of a nor-γ-eudesmenone (Figure 4). The dimethylbicyclo[4.4.0]decane framework was constructed starting from signals of hydrogens of methyl groups located on C4 and C10, respectively, and from the H7 (methine) hydrogen. The location of the acetyl group on C7 was confirmed by the connectivities of H12 with C7. Other heteronuclear connectivities were in agreement with the proposed structure. Moreover, the structure was confirmed by the proximity of the chemical shift values with those of γ-eudesmol and 10-*epi*-γ-eudesmol [21,22]. Unfortunately, the relative *cis* or *trans* stereochemistry of C10-Me and the acetyl group bore by C7 cannot be deduced from the through space H-H connectivities in the NOESY spectrum that appeared really poor. In both cases, the acetyl group should adopt an equatorial (or pseudo-equatorial) position. This point is confirmed by the multiplicity (doublet of triplets) and the coupling constant values (14.8 and 2.2 Hz) of equatorial H6 (^2^*J*_H6eq-H6ax_; ^3^*J*_H6-H7_ and ^4^*J*_H6eq-H8eq_, W stereochemistry) and it is in agreement with the occurrence of a correlation plot between H_6eq_ and H13 in the NOESY spectrum.

Comparison of the chemical shift values of B with those of the γ-eudesmol epimers reported in the literature could be informative. Indeed, the chemical shift of carbon C7 that differs drastically in γ-eudesmol (50.56 ppm) and *epi*-γ-eudesmol (44.21 ppm) [21,22] cannot be taken into consideration since compound **24** bore an acetyl group instead of the “isopropyl alcohol” group. However, a significant difference was observed on the chemical shift value of olefinic quaternary carbon C4, 124.48 ppm for γ-eudesmol and 125.98 ppm for 10-*epi*-γ-eudesmol, the latter being close to the C7 of compound **24** (126.29 ppm). The *trans* stereochemistry of the acetyl group and C10-Me was corroborated by considering the chemical shifts of carbons C1 and C9 of **24** (39.99 and 38.29 ppm) and comparing with those of *epi*-γ-eudesmol (38.13 and 39.46 ppm) and those of γ-eudesmol (40.22 and 42.28 ppm). Proton NMR also favors the *trans* stereochemistry. Indeed, C4-Me/C10-Me chemical shift values of **24** (1.67/1.06 ppm) fit perfectly with those of *epi*-γ-eudesmol (1.66/1.06 ppm) compared with those of γ-eudesmol (1.60, 1.01 ppm). Finally, the multiplicity (dt) and coupling constants (14.8 and 2.2 Hz) of H_6eq_ of **24** are in agreement with those of *epi*-γ-eudesmol (dt, 14.8 and 2.8 Hz) [21,22].

It should be mentioned that the C10 epimeric ketone has been reported by Marshall and Pike in the course of the stereoselective total synthesis of γ-eudesmol [23]. The relative *cis* stereochemistry of acetyl and methyl groups was deduced from mechanistic considerations and confirmed by MeLi addition to the keto group leading to γ-eudesmol. Although ^1^H-NMR data were not reported in detail, the chemical shifts of C4-Me and C10-Me (1.61 and 1.04 ppm) fit perfectly with those of γ-eudesmol (1.60 and 1.01 ppm), and therefore confirm the *trans* stereochemistry of C10-Me and acetyl group in **24**.

#### 2.3.3. Identification of 12-Hydroxy-isodihydroagarofuran (**39**)

**Figure 5:**
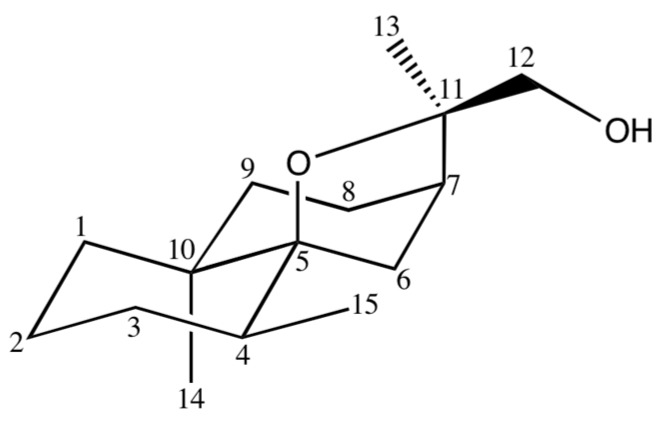
Structure of 12-hydroxyisodihydroagarofuran (**39**).

Sub-fraction F8.15, obtained by fractionation of F8 on silica gel, contained a main component (C, 60.1%; 2.3% in EO; RIa/RIp = 1747/2452). The ^13^C-NMR spectrum of that fraction exhibited 15 signals with high intensities. DEPT spectra allowed the differentiation of three C, two CH, seven CH_2_ and three CH_3_. Chemical shift values suggested the occurrence of an oxide function (2 C at δ = 88.21 ppm and 82.91 ppm) and a primary alcohol function CH_2_-OH (δ = 69.29 ppm) corroborated by the occurrence of two doublets at 3.40 and 3.24 ppm (^2^*J* = 10.4 Hz) in the ^1^H spectrum. The molecule contained also three methyl groups (^1^H-NMR signals: a doublet and two singlets). The formula C_15_H_26_O_2_ was deduced from these data in agreement with the molecular ion M^+^ = 238 in the MS. Obviously, the molecule displays a tricyclic structure. Owing to the occurrence of a CH_2_-OH group, one of the three rings is oxygenated. This suggestion was confirmed by examination of long range correlations in the HMBC spectrum. The bicyclo[4.4.0]decane skeleton was constructed starting from methyls C14 and C15 and the dihydroagarofuran framework resulted inter alia from correlations of H7 and H13 with various carbons (Table 3). The primary alcohol function was assigned to carbons C12 or C13 on the basis of the correlation plot of the methylenic hydrogens with C7. It was attributed to C12, the γ-shielding effect of the hydroxyl group on C13 being in the range 5–6 ppm, as expected. At this stage, component **39** should be a 12-hydroxydihydroagarofuran (Figure 5). Two points should be specified: (i) the stereochemistry of the ring junction of the decalin substructure; (ii) the relative stereochemistry of methyls C14 and C15. Unfortunately, the NOESY spectrum was particularly poor and therefore no significant through space interaction between hydrogen atoms has been detected. The correct stereochemistry was obtained by comparison of the ^13^C-NMR and ^1^H-NMR chemical shifts of **39** with those of the four dihydroagarofuran isomers reported in the literature [24,25,26,27]. Indeed, the ^13^C-NMR chemical shifts of C2 (21.33 ppm) and C4 (32.23 ppm) of **39** differ substantially from those of *trans*-dihydroagarofuran and 4-*epi*-*cis*-dihydroagarofuran (17.0/17.7 and 40.5/43.1, respectively) [25,27]. In contrast they are close to those of isodihydroagarofuran and *cis*-dihydroagarofuran (21.4/21.6 ppm and 32.2/32.6 ppm), respectively [24,26]. Differentiation between the last two isomers has been achieved by comparison of the ^1^H chemical shifts of methyls C14 and C15, 1.01 and 0.85 for **39**, much most closer to those of isodihydroagarofuran (1.00 and 0.88 ppm) than to those of *cis*-dihydroagarofuran (0.88 and 0.82 ppm). Therefore, compound **39** is 12-hydroxy-isodihydroagarofuran.

## 3. Discussion

Analysis of Vietnamese *Xanthocyparis vietnamensis* wood oil by a combination of chromatographic (GC, CC) and spectroscopic (GC-MS, ^13^C-NMR) techniques allowed the identification of 40 components that accounted for 87.9% of the oil composition. It is a sesquiterpene-rich oil whose composition is dominated by nootkatene (20.7%), a sesquiterpene hydrocarbon bearing the eremophilane skeleton and 11.12.13-tri-nor-eremophil-1(10)-en-7-one (17.2%). The eremophilane skeleton was also represented by valencene (3.5%), eremophil-9-en-11-ol (tr), eremoligenol (tr), dehydrojinkoheremol (0.2%), valerianol (tr), 13-nor-eremophil-1(10)-en-11-one (2.6%) and 11,12,13-tri-nor-eremophil-1(10)-en-7-ol (0.4%), the latter compound being found in Nature for the first time. Other components present at appreciable contents, including two new compounds, 10-*epi*-nor-γ-eudesmen-11-one and 12-hydroxyisodihydroagarofuran, displayed the bicyclo[4.4.0]decane framework of cadinane, eudesmane or selinane, Thirteen monoterpenes have also been identified (tr-4.1%; 14.8% in total).

The composition of *X. vietnamensis* wood oil varied drastically from those of leaf oils dominated either by hedycaryol (34.4%), phyllocladene (37.8%) (Edinburgh sample) [8] or by pimara-6(14)-15-diene (19.4%) and germacrene D (14.6%) (Vietnamese sample) [9]. Otherwise, nootkatene has been identified as a component of various essential oils, for instance Alaska cedar (*Chamaecyparis nootkatensis*) heartwood essential oil (20.1%) [28].

## 4. Materials and Methods

### 4.1. Plant Location and Essential Oil Isolation

Plant material (wood, about 4 kg) from *X. vietnamensis* has been collected in Quan Ba District. Hà Giang Province (Northern Vietnam, GPS coordinates: 23°09′N, 104°59′E, 1100–1200 m, Figure 1) and subjected to hydrodistillation for 3 h using a 5 L Clevenger-type apparatus yielding 8 mL of EO. The plant name was identified by Nguyen Sinh Khang by comparing morphological details. A voucher specimen has been deposited at IEBR under the reference: N.S. Khang 2014. A (HN).

### 4.2. Fractionation of Essential Oil

*X. vietnamensis* wood oil (2.2 g) was subjected to column chromatography (CC) on silica gel 250–500 µm, 35 g) using a gradient of solvents pentane/Et_2_O: 100/0 to 0/100. Ten fractions have been eluted: F1–F10. Fractions F2 (120 mg), F8 (466 mg) and F9 (345 mg) have been chromatographed once again (silica gel 250–500 µm) leading to sub-fractions F2.1–F2.6, F8.1–F8.18 and F9.1–F9.11 respectively. Sub-fraction F2.2 (18 mg, eluted with P:Et_2_O = 98:2) contained compound B = **24** (40.4%); sub-fraction F8.15 (43 mg, eluted with P:Et_2_O = 90:10) contained **39** (60.1%) and sub-fraction F9.7 (18 mg, eluted with P:Et_2_O = 80:20) contained **16** (52.5%). The three sub-fractions were subjected to spectroscopic analysis for structure elucidation.

### 4.3. Gas Chromatography (GC) Analysis

GC analyses were carried out with a Clarus 500 Autosystem (Perkin-Elmer, Courtaboeuf, France) apparatus equipped with two flame ionization detectors (FIDs) and fused capillary columns (50 m × 0.22 mm i.d. film thickness 0.25 μm). BP-1 (dimethylpolysiloxane) and BP-20 (polyethylene glycol). The carrier gas was helium with a linear velocity of 1.0 mL/min. The oven temperature was programmed from 60 °C to 220 °C at 2 °C/min and then held isothermal (20 min). Injector temperature was 250 °C (injection mode: split 1/60). Detector temperature: 250 °C.

### 4.4. Gas Chromatography-Mass Spectroscopy (GC/MS) Analysis

GC/MS analyses were carried out using an 7890A detector (quadrupole) (Agilent Technologies, Santa Clara, CA, USA) directly coupled to an Agilent Technology 5975C equipped with a fused-silica capillary column (30 m × 0.25 mm i.d. film thickness 0.25 μm). HP-MS 5% phenylmethylsiloxane. Carrier gas: helium at 1 mL/min; split 1:80; injection volume 0.2 μL. The injection port was set at 250 °C; the oven temperature was programmed from 60 °C to 250 °C at 4 °C/min (52.5 min). Significant quadrupole MS operating parameters: Ion source temperature 150 °C; electron impact ionization at 70 eV with scan mass range of 35–350 *m*/*z*.

### 4.5. ^13^C-NMR Analysis

^13^C-NMR analysis was performed on an AVANCE 400 Fourier Transform spectrometer (Bruker, Wissembourg, France) operating at 100.63 MHz for ^13^C equipped with a 5 mm probe in deuterated chloroform (CDCl_3_) with all shifts referred to internal tetramethylsilane (TMS). ^13^C-NMR spectra were recorded, at room temperature, with the following parameters: pulse width (PW) 4 μs (flip angle 45°); acquisition time 2.7 s for a 128 K data table with a spectral width (SW) of 24,000 Hz (240 ppm); CPD mode decoupling; digital resolution 0.183 Hz/pt. The number of accumulated scans ranged between 2000 and 3000 for each sample depending on the amount of oil available (18–40 mg in 0.5 mL of CDCl_3_). 2D NMR spectra were acquired using Bruker pulse programs.

### 4.6. Identification of Individual Components

Identification of the components was based on: (a) comparison of their GC retention indices (RI) on polar and apolar columns determined relative to the retention times of a series of n-alkanes with linear interpolation (Target Compounds software, version 6.3.2, Perkin-Elmer). with those of authentic compounds or literature data; (b) on computer matching with laboratory-made and commercial mass spectral libraries [14,29,30]; and (c) on comparison of the signals in the ^13^C-NMR spectra of essential oils with those of reference spectra compiled in the laboratory spectral library with the help of laboratory-developed software [10].

## Figures and Tables

**Table 1:** Chemical composition of *Xanthocyparis vietnamensis* wood oil.

No.	Components	RI	RIa	RIp	%	Identification
1	α-Thujene	932	922	1015	0.1	RI, MS
2	Sabinene	973	966	1124	tr	RI, MS, *^13^C-NMR*
3	Myrcene	987	979	1161	0.2	RI, MS, *^13^C-NMR*
4	1,4-Cineole	-	1003	1178	0.6	RI, MS, *^13^C-NMR*
*5*	*p*-Cymene	1015	1011	1271	1.3	RI, MS, ^13^C-NMR
6	Limonene	1025	1020	1202	0.2	RI, MS, *^13^C-NMR*
7	*p*-Cymenene	1075	1072	1436	2.4	RI, MS, ^13^C-NMR
8	*p*-Cymen-8-ol	1169	1159	1842	4.1	RI, MS, ^13^C-NMR
9	Terpinen-4-ol	1164	1161	1597	3.0	RI, MS, ^13^C-NMR
10	α-Terpineol	1176	1171	1690	0.5	RI, MS, ^13^C-NMR
11	Carvacryl methyl oxide	1226	1223	1602	0.2	RI, MS, *^13^C-NMR*
12	Carvacrol	1278	1275	2204	2.1	RI, MS, ^13^C-NMR
13	α-Terpinyl acetate	1335	1330	1685	0.1	RI, *^13^C-NMR*
14	β-Elemene	1389	1386	1588	0.8	RI, MS, ^13^C-NMR
15	11,12,13-tri-nor-Eremophil-1(10)-en-7-one	-	1438	1996	17.2	RI, MS, ^13^C-NMR
16	11,12,13-tri-nor-Eremophil-1(10)-en-7-ol (**A**)	-	1458	2173	0.4	RI, 2D-NMR
17	β-Selinene	1486	1480	1720	0.8	RI, MS, ^13^C-NMR
18	Valencene	1494	1487	1715	3.5	RI, MS, ^13^C-NMR
19	α-Selinene	1494	1490	1723	0.3	RI, *^13^C-NMR*
20	γ-Cadinene	1507	1492	1750	0.3	RI, *^13^C-NMR*
21	Nootkatene	1512	1507	1811	20.7	RI, MS, ^13^C-NMR
22	δ-Cadinene	1520	1512	1753	1.2	RI, MS, ^13^C-NMR
23	β-Elemol	1541	1532	2070	0.9	RI, MS, ^13^C-NMR
24	10-*epi*-nor-γ-Eudesmen-11-one (**B**)	-	1553	2039	0.9	RI, 2D-NMR
25	13-nor-Eremophil-1(10)-en-11-one	-	1594	2133	2.6	RI, ^13^C-NMR
26	Eremophil-9-en-11-ol (jinkoheremol)	-	1613	2206	tr	RI, *^13^C-NMR*
27	Eremoligenol	-	1616	2172	tr	RI, *^13^C-NMR*
28	γ-Eudesmol	1618	1617	2159	5.1	RI, MS, ^13^C-NMR
29	τ-Cadinol	1633	1625	2159	0.7	RI, ^13^C-NMR
30	τ-Muurolol	1633	1627	2176	0.7	RI, MS, ^13^C-NMR
31	δ-Cadinol (torreyol)	-	1629	2193	0.3	RI, *^13^C-NMR*
32	Valerianol	1647	1633	2207	tr	RI, *^13^C-NMR*
33	β-Eudesmol	1641	1634	2220	3.7	RI, MS, ^13^C-NMR
34	α-Cadinol	1643	1636	2220	1.9	RI, ^13^C-NMR
35	α-Eudesmol	1653	1639	2210	3.3	RI, MS, ^13^C-NMR
36	Selin-11-en-4α-ol	-	1643	2241	0.3	RI, *^13^C-NMR*
37	Cadalene	1659	1653	2204	0.3	RI, MS, *^13^C-NMR*
38	Dehydro-jinkoheremol	-	1671	2214	0.2	RI, *^13^C-NMR*
39	12-Hydroxy-isodihydroagarofuran (**C**)	-	1742	2245	2.3	RI, 2D NMR
40	Nootkatone	1782	1774	2501	4.7	RI, ^13^C-NMR
	**Total**				**87.9**	

Order of elution and percentages are given on apolar column. RI: retention indices from literature [14]. RIa, RIp: retention indices measured on apolar (BP-1) and polar (BP-20) columns, respectively. tr: traces (<0.05%); nd: not determined. *^13^C-NMR* (*italic*) = compounds identified in fractions of chromatography.

**Table 2:** NMR data of compound **24**.

C	δ C (ppm)	DEPT	δ ^1^H (ppm)	Multiplicity (*J*, Hz)	HMBC	COSY	NOESY
1	39.99	CH_2_	1.42 (a)	m^1^	-	-	-
			1.28 (b)	m	-	-	-
2	18.88	CH_2_	1.53	m	-	-	-
3	32.96	CH_2_	1.98 (a)	m	-	-	-
			1.86 (b)	m	-	-	-
4	126.29	C	-	-	-	-	-
5	131.73	C	-	-	-	-	-
6	25.89	CH_2_	1.96 (b)	m	C12	-	-
			3.01 (a)	dt (14.8;2.2)	C4; C5; C7; C8; C10	H12	H12; H13
7	49.02	CH	2.62	m	C5; C6; C8; C9	H12	H12
8	22.7	CH_2_	2.02 (a)	m	-	-	H8b
			1.84 (b)	m	C7; C9; C11/C5; C6; C10	H9	H8a
9	38.29	CH_2_	1.32	s	C1; C5; C7; C8; C10; C10-Me	H8b	-
10	34.57	C	-	-	-	-	-
11	211.19	C	-	-	-	-	-
12	28.06	CH_3_	2.14	s	C7; C11	H6a; H7	H7; H6a
C4-Me	19.55	CH_3_	1.67	s	C3; C4; C5; C10-Me	-	H6a
C10-Me	24.56	CH_3_	1.06	s	C1; C5; C9; C10	-	-

^1^ m = multiplet, s = singlet, d = doublet, t = triplet, (a), (b) = non equivalent protons.

**Table 3:** NMR data of 12-hydroxyisodihydroagarofurane (**39**).

C	δC (ppm)	DEPT	δH (ppm)	Multiplicity (*J*, Hz)	HMBC	COSY	NOESY
1	37.90	CH_2_	1.12 (a)	br s **^1^**	-		
			1.55 (b)	m	-		
2	21.33	CH_2_	1.44 (a)	t (3.0)	-		
			0.92 (b)	d (7.0)	C 15		
3	32.08	CH_2_	1.44 (a)	t (3.0)	-		
			1.35 (b)	m	-		
4	32.23	CH	1.74	m	C7	H15	
5	88.21	C	-	-	-	-	-
6	33.39	CH_2_	1.98 (a)	dd (11.7; 4.3)	C4; C5; C7; C8; C10	H6b; H8	H12a
			1.55 (b)	br s	C7; C8; C10; C11	H6a	
7	39.99	CH	1.87	m	C6; C8; C9; C12		
8	24.84	CH_2_	1.70	m	C9		
9	36.12	CH_2_	1.67 (a)	m	C14		
			1.20 (b)	m	C8		
10	38.83	C	-	-	-	-	-
11	82.91	C	-	-	-	-	-
12	69.29	CH_2_	3.40 (a)	d (10.4)	C7; C11; C13	H12a; H13	H6a; H15
			3.24 (b)	d (10.4)	C13	H12b; H13	
13	17.76	CH_3_	1.41	s	C7; C11; C12		
14	23.69	CH_3_	1.01	s	C1; C5; C9; C10		
15	15.52	CH_3_	0.85	d (7.0)	C3; C4; C5	H4	

^1^ br s = broad singlet, d = doublet, t = triplet, m = multiplet, (a), (b) = non equivalent protons.
